# Application of discrete event simulation in health care: a systematic review

**DOI:** 10.1186/s12913-018-3456-4

**Published:** 2018-09-04

**Authors:** Xiange Zhang

**Affiliations:** Department of Health Care Management, Institute of Public Health and Nursing Research, Health sciences, University of Bremen, Grazer Str. 2a, 28359 Bremen, Germany

**Keywords:** Systematic review, Discrete event simulation, Health care management

## Abstract

**Background:**

The objective was to explore the current advances and extent of DES (Discrete Event Simulation) applied to assisting with health decision making, as well as to categorize the wide spectrum of health-related topics where DES was applied.

**Methods:**

A systematic review was conducted of the literature published over the last two decades. Original research articles were included and reviewed if they concentrated on the topic of DES technique applied to health care management with model frameworks explicitly demonstrated. No restriction regarding the settings of DES application was applied.

**Results:**

A total of 211 papers met the predefined inclusion criteria. The number of publications included increased significantly especially after 2010.101 papers (48%) stated explicitly disease areas targeted, the most frequently modeled of which are related to circulatory system, nervous system and Neoplasm. The DES applications were distributed unevenly into 4 major classes: health and care systems operation (HCSO) (65%), disease progression modeling (DPM) (28%), screening modeling (SM) (5%) and health behavior modeling (HBM) (2%). More than 68% of HCSO by DES were focused on specific problems in individual units. However, more attempts at modeling highly integrated health service systems as well as some new trends were identified.

**Conclusions:**

DES technique has been an effective tool to approach a wide variety of health care issues. Among all DES applications in health care, health system operations research occupied the most considerable proportion and increased most significantly. Health Economic Evaluation (HEE) was the second most common topic for DES in health care, but with stable rather than increasing numbers of publications.

## Background

DES is a form of computer-based modeling methodology that provides an intuitive and flexible method [1], characterized by the ability to simulate dynamic behaviors of complex systems and interactions between individuals, populations and their environments [2]. The goal of such a model is to comprehensively compare potential practices or strategy options, so as to identify the most efficient and effective ones, especially in situations where it may be infeasible to carry out required surveys or comparative experiments in practice.

In comparison with aggregate models without interaction [3] like decision trees or Markov models, DES as an operational research technique can be more advantageous to model complex systems at the individual level instead of cohort level. The contexts simulated are usually viewed as queuing networks where individual entities pass through a series of discrete events one by one at discrete intervals, between which they have to wait in queues due to the constrained availability of resources [4]. DES is also able to allow decision makers to conduct “what if” analyses by changing the operational scenarios and rules, to predict the possible impacts resulting from a variety of policy alternatives before truly translated into practice without any alteration in present systems. However, admittedly DES models are also simplified presentations of reality, just as the portraits of other modeling techniques indicate. Typically, patients or medical care providers are referred to as entities, an essential concept of DES, which go through the whole process simulated. Attributes of target entities, which can affect or even determine entities’ responses to events, include age, sex, health status, illness history, duration of disease and other demographics, the values of which are updatable as models are running [1, 5]. Events can be viewed as a wide range of things which may happen during simulation, like the occurrence or recurrence of a disease, admission to health facility, delivery of some medical treatment or transitions between health states. More detailed characterizations of DES are available from the studies by Caro and Karnon et al. [5, 6].

There are two relatively extensive reviews on DES applications in the health care sector [7, 8], which were restricted to specific sub-systems or contexts of health care systems. The earlier one was published in 1999, surveying and classifying the DES literature which focused on single health care units [7]. Around ten years later, the second one sought to analyze DES applications in hospital care [8]. To the best of our knowledge, the overall picture of how DES has been applied to health care is still unclear.

The aim of this review is to provide an up-to-date overview of a considerable body of academic journal literature relevant to the application of DES in health care, thereby further gaining deep insights into the extent of DES used to assist with health decision making and categorizing the wide array of health-related topics where DES was applied.

## Methods

### Search strategy

For this systematic review, two main databases, PubMed and Web of Science, were systematically searched as the main sources for publications of interest. The last search was performed on March 31, 2017, based on broad search terms: ‘discrete event simulation’ or ‘(discrete event) AND (computer simulat* OR model*)’, accompanied by terms like ‘health service*’, ‘Patient*’, ‘healthcare’ or ‘health care’. Papers which brought up discrete event simulation and health-related topics in the title, abstract or keywords and were published over the last 2 decades were searched for without any restrictions on setting and country of origin applied. As a supplement, the reference lists of relevant reviews previously published and the latest studies included were scanned and hand-searching of pertinent open-access journals was also conducted in order to retrieve additional potential peer-reviewed publications.

### Inclusion criteria

To identify studies pertaining to our intentions, the inclusion of papers was based on the following criteria:

- Studies had to be carried out in health care delivery or public health scenarios, which can be related to any specific context of health service systems.
- Discrete event simulation frameworks should be pointed out as the main modeling technique and independently formulated and structured.
- How exactly work flows were structured and simulated should be clarified and clearly demonstrated in the form of either flow charts or descriptive texts, according to the recommendations of the ISPOR-SMDM modeling good research practice [1].
- Only peer-reviewed original research was included for further review. Papers published as literature reviews, editorials, conference proceedings or methodological guidelines were excluded.
- Only English articles were taken into account.

### Selection of publications

After removing duplicates, all studies were screened and reviewed following two main phases. The initial stage of this review was conducted independently by XG and FG following the predefined criteria mentioned above, at title and abstract level, with the aim to identify studies which are appropriate for further full-text review. For inclusion criteria 1, 2 and 4, titles and abstracts screened in the Microsoft Excel table provided adequate information for reviewers to make decisions, but for criterion 3, full texts of records were to be retrieved, when necessary. In case of discrepancies in assessment results between the two reviewers, a third reviewer (WR)’s opinion was taken into consideration.

In view of the emphasis of this review laid on the overview of application advances of DES in health care, therefore only basic information hints of each paper retrieved were deemed necessary to be extracted and screened on a standardized Excel form. All papers were stored and managed via EndNote X7. The following items were extracted and summarized from the literature: Title, author, publication date, journal, medical conditions, perspective for cost measurement, specific settings analyzed, setting classification and application category, on the latter two of which the two precedent similar reviews were focused respectively [7, 8]. In this sense, this work could be perceived as an update for our precedents. Nonetheless, more detailed insights can be gained herein, for example, this review presented the physical contexts simulated more specifically instead of only roughly classifying them as inpatient facilities, outpatient clinics and emergency department. Additionally, a more comprehensive taxonomy of application areas was introduced, which was built on reviews by Mielczarek et al. [9] and Fone et al. [10]. To indicate the breadth and scope of DES applications in health care, medical conditions surveyed were also indicated and categorized based on ICD-10 codes (International Statistical Classification of Diseases and Related Health Problems 10th Revision) endorsed and recommended by WHO.

## Results

### Search results

A total of 479 citations were yielded in the first step. After removal of 123 duplicates, 356 papers were screened and reviewed at title and abstract level. This first round of reviewing resulted in the exclusion of 136 articles which failed to meet the inclusion criteria: 8% of articles excluded were not related to health care domain, 38% were not original studies, 34% did not perform DES independently, 19% did not or not clearly present DES structures and entity flows and only one paper was not in English.

**Fig. 1:**
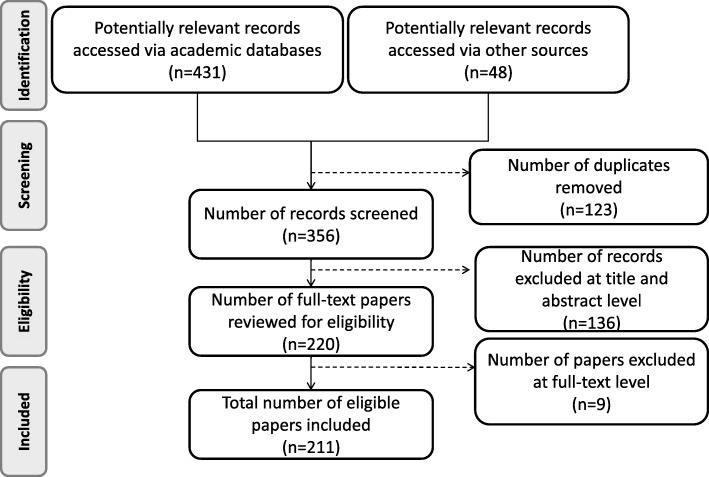
Flow chart of this systematic review

The remaining 220 articles were further reviewed on the full text level. During this phase, the final 211 articles sufficiently met the eligibility criteria and 9 were excluded mainly due to the fact that they were methodological analyses instead of original research, 2 of which actually focused on other modeling techniques, not obviously related to DES. The review process and results are illustrated as a PRISMA diagram in Fig. 1.

### Growing trend of DES applications in health care

**Fig. 2:**
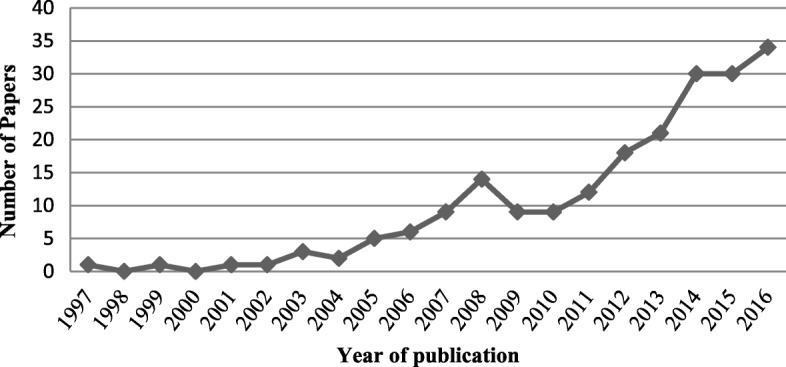
Number of studies included in each year of publication

The last two decades witnessed a noticeable scale of expansion of publications in this arena as illustrated in Fig. 2, especially after 2010. The number of publications increased from less than 10 papers annually published before 2010 (except for 2008) to almost 35 in 2016, with 73% of the included papers published after 2010. This indirectly reflects a constantly rising popularity of DES modeling in health care management.

### Categorization of applications

**Fig. 3:**
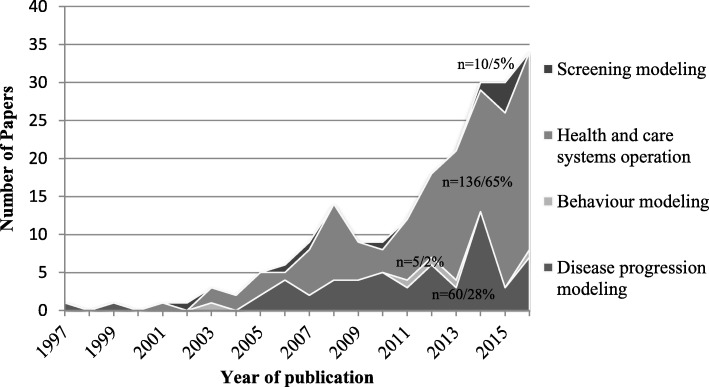
Categorization of applications of DES in health care

Based on the nature and inherent properties of the DES models selected, they can be arguably classified into 4 major categories, which were inspired by previous taxonomy [9, 10]: health and care systems operation (HCSO), disease progression modeling (DPM), screening modeling and health behavior modeling (HBM) (Fig. 3), which are explained further in the subsequent sections. Figure 3 illustrates that health and care systems operation accounts for the most considerable proportion (65%) of all modeling studies throughout the whole time span, followed by disease progression modeling (28%), relative to the limited proportion of screening (5%) and health behavior modeling (2%). In addition, the number of health and care systems operation modeling increased by the largest margin compared to other topic areas and disease progression modeling remained at around 5 publications level per year. What is also worth noting is that modeling screening programs and health behavior changes by DES seems to have drawn the attention of health care modelers in recent years.

### Disease distribution

**Fig. 4:**
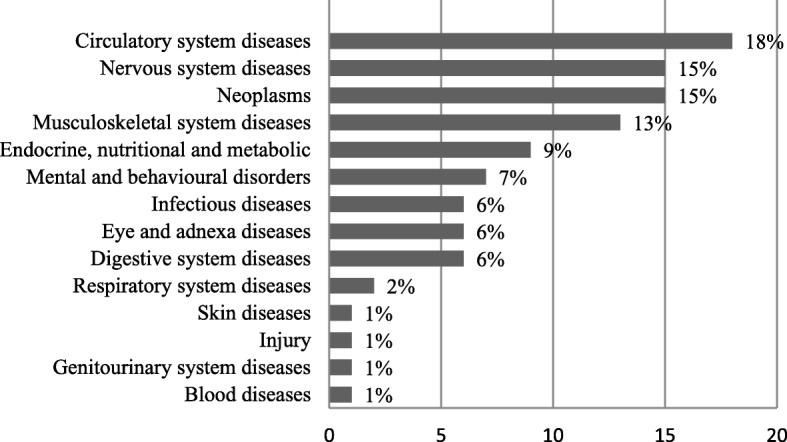
Distribution of disease areas analyzed in studies

Research which specified a naturally developing history of a specific disease and an according course of treatment was classified in terms of medical conditions in this section. Out of the 211 papers included, there are a total of 101 papers (48%) which stated explicitly that specific disease areas and corresponding treatment flows were targeted and modeled. The most frequently analyzed medical indications are related to circulatory system, nervous system, Neoplasm and musculoskeletal system diseases, occupying 18%, 15%, 15% and 13% respectively. The detailed distribution of diseases focused on is clearly displayed in Fig. 4.

ICD-10 is divided into 22 chapters from chapter I to chapter XXII, each of which can be further divided into a number of sub-categories, and chapters I-XVII are associated to specific morbid conditions and different body systems. This distribution of diseases presented in Fig. 4 covers more than half (14) of all 22 chapters of ICD-10. Furthermore, it is worth noting that 13 (76%) of all the 17 chapters I-XVII of ICD-10 and nearly all categories related to body systems, except for chapter VIII (Diseases of the ear and mastoid process), are included in Fig. 4 to varying degrees.

### Disease progression modeling

In regard to disease progression modeling, DES is commonly employed to transparently conceptualize and construct the course of diseases, health states transition and disease-related events patients will go through under different interventions [11, 12]. This application field is mainly intended to assist in making well-informed medical decisions through comparing different treatment alternatives at medical level in terms of resources consumed, health outcomes or both.

Forty six (77%) of all 60 disease progression modeling compared various medical interventions from a cost-effectiveness perspective. Ten (16%) were undertaken in order to find the most beneficial treatment options, based only on either economic cost or health impact dimension. Most of the remaining 7% papers were predictive models for future health service demands. In total, the most (56) of disease progression models are related to health economic evaluations.

Of the 51 papers which collected and analyzed cost data, 21 (41%) employed the health care payer perspective. 15 (29%) and 14 (28%) were conducted from the perspective of the health care system and society, respectively, with only one analysis taking the point of view of a health care provider.

### Health and care systems operation

Health and care systems operation is meant to enable health care managers to better understand the underlying mechanisms of how a system operates, comprehensively investigate complex relationships among different sections within a system with the aid of DES, so as to make optimal operational and administrative decisions [9, 13]. As has been presented in section “Categorization of applications”, DES is most commonly utilized for operations research purposes in health care, with the most significant upward trend in contrast with other application areas.

#### Distribution of settings for HCSO

It has been repeatedly pointed out that the applications of DES were highly confined to specific single health care units with rare attempts to model broader scenarios [7, 8]. The results of this review are also in support of this conclusion. As is presented in Table 1, more than 68% (93) of HCSO concentrated on single micro-systems. Furthermore, consistent with previous reviews and surveys [8, 14, 15], the major attention of health care modelers was mainly focused on modeling workflows of emergency departments (32) and intensive care units (10) relative to other health care contexts modeled.

**Table 1:** Distribution of settings of HCSO by DES

Hospital-based outpatient clinic	26/19.1%	Inpatient department	7/5.1%
Orthopedics	6/4.4%	Orthopedics	4/2.9%
Pain	3/2.2%	Cardiovascular	2/1.5%
Endoscopy	2/1.5%	Obstetrics	1/0.7%
Obstetrics	2/1.5%	Multi-facility health care provider	18/13.2%
Procedure center	2/1.5%	Hospital	12/8.8%
Physiotherapy	2/1.5%	Primary care clinic	3/2.2%
Cardiology	1/0.7%	Blood center	1/0.7%
Dermatology	1/0.7%	Obesity care center	1/0.7%
Vaccination	1/0.7%	Sleep center	1/0.7%
Neurology	1/0.7%	Emergency department	32/23.5%
Fetal diagnosis and treatment	1/0.7%	Intensive care unit	10/7.4%
Phlebotomy service	1/0.7%	Operating theater	7/5.1%
Dental clinic	1/0.7%	Auxiliary department	11/8.1%
Ophthalmology	1/0.7%	Multi-departmental network	7/5.1%
Teaching ambulatory care	1/0.7%	Health care system	18/13.2%

*n*/%:Number of studies/proportion of studies in all HCSO papers

Nevertheless, the potential of DES for representing broader continuums of health care delivery system was increasingly taken advantage of. Multi-facility health service providers and even health care systems as a whole were more frequently modeled by DES than reported before, both taking up around 26% (36) of all HCSO papers. The remainder 6% examined coordination effectiveness among departments or micro-systems within a certain health service provider.

#### Sub-categories of HCSO

**Fig. 5:**
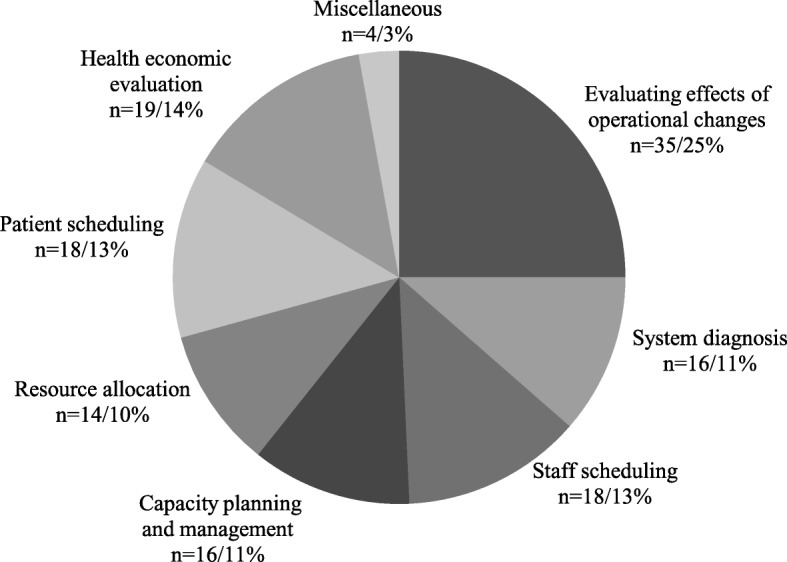
Distribution of sub-categories of HCSO

The DES approach has been used to address a wide range of operational challenges encountered by health care stakeholders, as is depicted in Fig. 5. This taxonomy was derived from the proposal of Lagergren [13], which was extended in this review by adding the category of “health economic evaluation”. This classification method distinguishes six major operations research issues consisting of patient scheduling (including appointment and discharge scheduling for both outpatient and inpatient care), resource allocation, capacity planning and management, staff scheduling, system diagnosis and evaluating the effects of operational changes or reconfigurations, the last one of which occupies the most substantial proportion (25%) of all HCSO papers and all others stand at a very similar level of around 12%. 19 (14%) health economic evaluations were identified in HCSO category.

### Screening modeling

A total of 10 screening modeling papers by DES were identified in this review, most of which were published after 2010.

Among all DES screening models, cancer screening modeling has been most frequently embarked on. It is noteworthy that breast cancer screening dominates this type of modeling, accounting for 50% of cancer models. DES was applied to investigate the costs and health outcomes of different mammographic follow-up schedules [16, 17], alternative breast cancer screening programs [18, 19] as well as the routine performance of a mammography facility under different operational conditions [20]. Health benefits were commonly measured as gains in QALY (quality-adjusted life years) and costs were mainly measured from the perspective of the health care payer and system.

Other screening studies reviewed incorporate two models which analyzed the effects of distinct colorectal cancer screening strategies on patient outcomes and system performance [21, 22], with the remaining evaluating different screening practice options for tuberculosis [23], Niemann–Pick type C disease [24] and diabetic retinopathy [25].

### Health behavior modeling

In marked contrast to other industries modeled by DES, the health care domain is deemed more complex to simulate. One important reason, among others, is that entities such as patients or health service providers modeled are individuals characterized by complicated psychological and behavioral attributes. This attribute makes health care more difficult to be represented by computer simulation than to purely model production machines or materials. Incorporating human behavioral factors into modeling practice is a newly emerging concern for health care modelers. Most studies of this type were published after 2011.

One attempt for health behavior modeling was to view a certain individual behavior as the object to be modeled, in others words, to model the trajectories of individual behavioral changes over time under different interventions in order to determine the most influential and efficient mechanisms to encourage desirable health behaviors. This category was highly dominated by smoking-cessation behavior. Smoking behaviors, quit attempts, relapses and sets of events corresponding to smoking-cessation behaviors were simulated by DES structures with the intention to identify the most cost-effective smoking-cessation strategies for diverse populations [26–28].

There were other attempts to take human behavioral factors into account while modeling health care delivery, so as to better reflect what really happens in reality. The latest example is the model developed by Brailsford et al. [29], which represented screening policies for breast cancer via DES with the addition of behavioral factors to each simulated patient’s characteristics. The earliest case was published in 2003 and presented a screening model for diabetic retinopathy focusing on attendance behavior through embedding individual emotion, cognition and social status elements [30].

## Discussion

This systematic review was undertaken to summarize the advance status and new trends of the application of DES in health care over the last two decades and provide a more quantitative overview of a larger number of DES models compared to precedent reviews.

### Rarely applied at policy strategic level

The extent to which DES modeling has been employed to deal with issues at the strategic level is relatively disappointing, which is possibly due in part to the intrinsic attribute of this modeling technique. According to Mielczarek et al. [9], the DES framework lends itself better to simulate operational details and physical mechanism of a specific process instead of health policy evaluation and planning. Thus, DES is a suitable tool when it comes to concrete operational issues of health care delivery. Regarding public health policy evaluation, in contrast, System Dynamics (SD) is viewed as a more preferred modeling technique.

However, there are still some efforts of applying DES on this highly aggregate level. One of these is the study conducted by Deo et al. [31], which evaluated the long-run merits and drawbacks of integrating outpatient and HIV services in sub-Saharan Africa. Nguefack et al. built a DES model to estimate the number of new pediatric HIV infections through Mother-to-Child Transmission [32].

### More efforts towards modeling integrated multi-facility providers were identified

The majority of modeling studies included are unit specific, focusing on individual micro-systems such as emergency department and intensive care unit, consistent with previous reviews [7, 8]. Nonetheless, there are an increasing number of studies modeling complex integrated healthcare providers, which were published particularly after 2010. Certainly, these independent multi-facility providers modeled as a whole tended to be simulated at an aggregate and abstract level, because it appeared infeasible to model in great detail—only a sequence of essential activities occurring during patients’ stay from admission to discharge were represented by DES frameworks.

Health care management could benefit a lot from DES used to model integrated providers as a whole rather than just limiting to single units. Given the intricate interdependencies of modern health care services, a more realistic representation of the workings of a real system will be achievable from this integrative perspective. Instead of simplifying the multi-faceted treatment pathways of acute bacterial skin and skin structure infections (ABSSSIs), Revankar et al. [33] developed a DES framework which captured the complete variations in locations of care from emergency departments, through to outpatient and inpatient units, which properly reflected the real-world complexities related to such treatment administrations.

High-level decision supports can be provided via DES models for hospital managers in terms of the diagnosis of system inefficiencies and evaluation of alternative system configurations [34–37]. Patient flow through a multidisciplinary sleep center was constructed by Pendharkar et al. with the support of DES [36]. Multiple system constraints resulting in treatment access delays were recognized and alternative reconfigurations aimed at improving access to sleep care were tested.

Patient satisfaction lies in their comprehensive experience throughout a whole integrated system. Consequently, to model an integrated provider as a whole is considered necessary for improving patient overall experience [38]. Bard et al. [39] applied DES to mimic patient flow through a series of activities as a network in a Family Health Center and gained deep insights into the influential factors jeopardizing patient satisfaction such as excessively long hospitalization and high provider utilization.

Certainly there are a few pitfalls for DES modeling at this higher lever in health care. The most frequently mentioned would be the generalizability issue. These model frameworks for integrated care providers were usually populated by data collected from specific institutions during a certain historical period, which in this sense leads to limitations on the applicability of modeling results into other settings or other periods of time [34, 36]. In addition, lack of necessary data makes it inevitable to make assumptions about the systems modeled or rely on expert estimates as well as data from literature instead of real observations. These alternatives naturally can help simplify the DES modeling practice, but, how and to what extent the validity and accuracy of DES models will be affected should not be disregarded [33, 40, 41].

### Modeling of the continuum of entire health services for a certain disease area emerged

Health service pathways are a continuous process throughout the whole health system involving a wide range of care providers, from primary health care clinics (downstream), to highly specialized tertiary units (upstream), caring for different phases of disease progression. Unexpectedly, a few DES models of this continuum of care have been reported since 2005, imitating the integrated networks and interconnectedness among different parts of the whole health care pathways for a certain disease area.

The exploration of DES in this direction started in 2005, which, since then, opened up the possibilities of drawing the attention of health care modelers to a bigger picture of the relationships between diverse components of a health care continuum. These macro modeling endeavors are intended to deal with some critical challenges in which micro-system models can hardly play a role, for example, evaluating the cooperation and transferring efficiency [42, 43] and balancing the distribution of scarce health resources within a health system [44].

Medical and administrative initiatives may be favorable for one specific section of a health system where they will be conducted, but may conversely cause unintended damages to other parts, or lead bottlenecks to shift within the system [42, 43, 45]. Therefore, applying DES to evaluate the overall long-term performance of policy changes from a scale perspective is considered appealing. With the assistance of DES in modeling the care system for Parkinson’s disease (PD) in the UK, Lebcir et al. [42] reported the impact of an increased use of PD community services on the demand for hospital-based healthcare and resources.

In addition, complex health care interventions nowadays usually imply integrative programs, implementations of which require the deployment of profound organizational and systematic changes and improvements. Accordingly, analyzing the potential benefits of such sophisticated interventions necessitates the employment of a systematic and holistic horizon [46–49]. As an example, a recent research presented by Willoughby et al. [48] mapped a system-wide patient flow with DES through a health service system for speech language pathology and further tested the effectiveness of integrated policy changes which involved increasing group treatments, paraprofessional utilization and so on.

Similarly to the shortcomings stated in section “More efforts towards modeling integrated multi-facility providers were identified”, these system-wide DES simulations also suffer limited generalizability and lack of sufficient data needed. In addition, the construction of macro-models is considered far more time-consuming and data-intensive because of higher complexity and vast numbers of activities involved [43]. Furthermore, synthesizing of data from a range of distinct sources will lead to additional uncertainty into parameters estimated [50]. Another challenge mentioned by Katsaliaki et al. [51] is the lack of consistent terminology. The same processes or services may be named quite differently across different parts of a health system, which can also give rise to some difficulties in modeling.

### Advantages of DES in health economic evaluation have been increasingly admired

According to the results of this review, health economic evaluation is the second most common theme, occupying 36% (75) of all DES applications in health care, following health operations research.

Although Markov models and decisions trees are still the most frequently applied modeling techniques in pharmacoeconomic evaluations, it is not hard to find the arguments for the advantages of DES used in HEE that highlight the valued potential of DES to overcome the inherent limitations of cohort Markov models and decision trees.

One merit of DES is called “individual heterogeneity”, namely, each of the entities modeled is stochastically assigned their own unique properties at the beginning of simulation [26, 52–54]. Moreover, these initial characteristics can be updated according to events experienced over time, which will in turn impact the possibilities of the occurrence of following events [55]. Hartz et al. [52] argued that individual characteristics neglected by most of cohort models might have profound impacts on the long term outcomes predicted. The omission of individuality could be viewed as an obvious deficiency for homogenous cohort models.

Another noteworthy point of DES for HEE is its allowing for “individual interaction”, which is valuable especially for models involving analyzing the effects of treatment delays due to patient competition for constrained resources [56, 57]. Standffield et al. [56] conducted a cost-utility analysis using DES because of the need to consider the impact of introducing OPSC (Orthopaedic Physiotherapy Screening Clinics and Multidisciplinary Services) on patients queuing time.

Additionally, the advances of the DES-specific software packages make it possible to communicate the frameworks and results of HEE to lay decision makers in an easier and more intuitive way, for example with different levels of animation [58]. The transparent presentation plays an important role in improving the understandability and acceptability of model outputs to health care stakeholders [5, 59, 60].

There are also other reasons for DES being chosen in preference to other alternatives, such as less limitations on the number of events and health states to be represented [61, 62], higher flexibility, less computing intensity [63], and event-based conceptualization attribute [55]. Taken together, DES models are increasingly considered as a more practical and flexible modeling technique in case of complex and dynamic contexts in health care.

### Limitations

There are several limitations of this review which need to be mentioned. Firstly, only PubMed and Web of Science were searched as main databases. This seems to limit the supply of target publications. However, given the significant number of studies included and no novel application areas of DES in health care was further identified in the later reviewing stage, it is reasonable to believe that the majority of representative literature has been captured.

Secondly, the inclusion criteria could also be seen as a limitation. Only peer-reviewed original DES studies in health care management, which unambiguously presented model structures and mechanisms, were given consideration. This set of criteria excluded a certain proportion of literature, especially papers compiled in proceedings of prestigious conferences. Nevertheless, these criteria were deemed necessary in order to make sure the modeling techniques employed in papers were indeed DES frameworks and also ensure consistency with the suggestions by ISPOR-SMDM [6]. In addition, as discussed before, it was believed that the sufficient representativeness of the papers included was not affected substantially in this review.

Thirdly, this review is limited to a broad overview of applications of DES in health care delivery, but without critically assessing their reporting quality. There is thus a need for more detailed quantitative analyses which were beyond the scope of this study.

## Conclusion

This systematic review has presented the versatility and new trends of DES models applied to deal with a broad spectrum of issues facing health care administrators and stakeholders. A survey of 211 studies was conducted and the rapidly growing popularity of DES in health care sector could be reflected by the substantially and constantly increasing number of publications, particularly after 2010. In addition, this extending trend could also been proven by the fact that more than half of ICD-10 chapters have been covered by DES studies. This could probably be viewed as an indication that DES models are becoming more and more embraced in health care management, but this point warrants further evidence of the value and extent of DES implementations in realistic health care decision making, which would be one of the focuses of the following publication.

In this survey, DES models were arguably classified into 4 main categories based on the nature of subjects modeled. This classification may need more discussions because of the intrinsic complexity and overlapping of the applications of DES in health. Undoubtedly, health and care systems operations were the dominant issues approached with the assistance of DES, occupying a massive proportion and expanding at the highest rate. The dominance of this leading application area has also been demonstrated in previous comprehensive reviews on computer simulation in health care, especially, the reviews conducted by Mielczarek et al. [9] and Fone et al. [10] which evidently presented quantitative supporting evidence for the extensive use of DES in health system operations.

Although modeling of specific individual units still has a predominant role in all the HCSO areas, more scientific endeavors to simulate more integrated health care settings like hospitals or even the whole health service continuum have been identified than before, which implies the rich potential of DES to provide a broader picture of how health care systems behavior. Both of the precedent DES reviews by Jun et al. [7] and Günal et al. [8] also discussed the weaknesses of unit-specific piecemeal modeling as well as the significance of modeling at the macroscopic and integrated level. Additionally, the causes and barriers of the limited number of higher-level DES models were also analyzed and delineated in both.

Health economic evaluations via disease progression modeling have become the second most common theme among all DES applications in health care. This topic area was distinguished as one of major categories in the classification systems proposed by Fone et al. [10] and Katsaliaki et al. [64]. However, no significantly increasing trend in this sphere has been discovered. The majority of these evaluations could be reasonably categorized as cost-effectiveness analysis, approximately half of which were carried out from the perspective of health care payers.

It is hoped that health policy developers could yield an up-to-date overall picture of the application of DES across various health-related topic areas and get motivated to actively apply DES techniques to tackle the conundrums they are facing. Regarding the quality and value of the research surveyed, a critical appraisal will be demonstrated in the following publication.

## Abbreviations

ABSSSIs: Acute Bacterial Skin and Skin Structure Infections
DES: Discrete Event Simulation
DPM: Disease Progression Modeling
HBM: Health Behavior Modeling
HCSO: Health and Care Systems Operation
HEE: Health Economic Evaluation
ICD-10: International Statistical Classification of Diseases and Related Health Problems 10th Revision
ISPOR: International Society for Pharmacoeconomics and Outcomes Research
PD: Parkinson’s Diseases
PRISMA: Preferred Reporting Items for Systematic Reviews and Meta-Analyses
QALY: Quality-adjusted Life Years
SD: System Dynamics
SM: Screening Modeling
SMDM: Society for Medical Decision Making
